# Butyrate mitigates multi-organ damage in urosepsis: associated changes in the renal NOX4/ROS/NF-κB axis and gut microbiota

**DOI:** 10.3389/fcimb.2026.1912506

**Published:** 2026-09-01

**Authors:** Yan Liu, Zhenyu Liu, Hao Xu, Yu Zhou, Yafeng Chen, Lin Wang, Fuhan Zhao, Lei Gao, Tiejun Pan

**Affiliations:** 1 Department of Urology, General Hospital of Central Theater Command Graduate Joint Training Base, School of Medicine, Wuhan University of Science and Technology, Wuhan, Hubei, China; 2 Department of Urology, General Hospital of Central Theater Command of Chinese People’s Liberation Army, Wuhan, Hubei, China; 3 School of Clinical Medicine, Hubei University of Chinese Medicine, Wuhan, Hubei, China; 4 General Hospital of Central Theater Command and Hubei Key Laboratory of Central Nervous System Tumor and Intervention, Wuhan, Hubei, China

**Keywords:** butyrate, gut microbiota, organ damage, oxidative stress, urosepsis

## Abstract

**Introduction:**

Urosepsis is a kind of sepsis resulting from urogenital infections, marked by a significant prevalence of organ failure and mortality. This study sought to examine the preventive effect and possible mechanism of butyrate against multi-organ damage caused by urosepsis.

**Methods:**

This work utilized a ureteral obstruction model complicated by infection to more precisely replicate acute kidney injury and the systemic inflammatory response associated with urosepsis. Rats received butyrate supplementation or antibiotic therapy following modeling, multi-organ function, histopathology, inflammatory markers, renal oxidative stress levels, and gut microbiota composition were comprehensively assessed.

**Results:**

The findings indicated that in rats with urosepsis generated by ureteral obstruction, renal NOX4 was markedly elevated, the inflammatory response was exacerbated, and various organ dysfunction was compromised. Butyrate intervention markedly diminished blood levels of TNF-α, IL-6, and MCP-1, and dramatically downregulated kidney expression of KIM-1 and NGAL, as well as macrophage infiltration. Moreover, butyrate significantly mitigated cardiac mitochondrial injury and raised liver transaminases, enhanced the expression of intestinal ZO-1 and occludin proteins, and restructured the gut microbiota. Mechanistic studies demonstrated that butyrate treatment was associated with reduced renal NOX4 expression, reduced ROS levels, and decreased phosphorylation of IκBα and p65, together with attenuated renal inflammation and multi-organ injury. These effects may help restrain the spread of kidney-derived inflammation and, in turn, alleviate multi-organ injury. Concurrently, by altering the gut microbiota and enhancing the growth of beneficial intestinal bacteria, it further elevates the synthesis of short-chain fatty acids in the gastrointestinal tract. Furthermore, by incorporating a combined butyrate and antibiotic treatment group, we observed that multiple physiological indicators were substantially improved in the combination therapy group compared with either the butyrate monotherapy group or the antibiotic monotherapy group, confirming that the concomitant administration of butyrate and antibiotics exerts a synergistic therapeutic effect.

**Conclusions:**

In a urosepsis model caused by urinary tract obstruction and infection, butyrate attenuated renal and systemic inflammation and multi-organ injury, accompanied by reductions in renal NOX4 expression, ROS levels, and NF-κB activation, consistent with modulation of the renal NOX4/ROS/NF-κB pathway. These findings highlight the potential therapeutic value of butyrate as an adjunct to antibiotic therapy.

## 1 Introduction

Sepsis is characterized by the international medical community as a life-threatening illness of multiple organ dysfunction (MODS) resulting from a dysregulation of the host’s inflammatory response to infection. Sepsis results in around 11 million fatalities each year, representing 20% of all global deaths. Urosepsis, originating from urinary tract infections (UTIs), constitutes 20% to 40% of all sepsis cases, with mortality rates soaring to 25% to 60% among the elderly, immunocompromised individuals, and specific populations with urinary tract blockage (Tandogdu et al., 2024; Kranz et al., 2024). The high prevalence of UTIs in the population, coupled with the rising incidence of complex UTIs attributable to factors such as aging, diabetes, urinary stones, indwelling catheters, and urinary tract interventions, renders urosepsis a significant concern at the nexus of critical care medicine and urology. Clinical investigations indicate that patients with urosepsis frequently exhibit organ dysfunction at the time of diagnosis (Tandogdu et al., 2024). It is crucial to highlight that urosepsis is not merely an intensification of a localized infection; its fundamental concern is the life-threatening organ dysfunction caused by the infection inducing a dysregulation of the host response (Wagenlehner et al., 2015). In urinary tract infections, the kidneys frequently serve as the initial site of the pathological process: upper urinary tract obstruction causes a significant rise in intrarenal pelvic pressure, enabling local pathogens (primarily *Escherichia coli*) and endotoxins to penetrate the compromised renal pelvic venous fornix barrier, leading to retrograde bloodstream infection. The elevated intrarenal pelvic pressure and bacterial burden collaboratively influence the renal tubular epithelium and renal interstitial milieu, prompting immune cell infiltration, cytokine secretion, microcirculatory disruptions, and the accumulation of oxidative stress (Hong et al., 2023). Upon experiencing acute injury, the kidneys’ difficulties extend beyond the mere deterioration of renal function. Distant organs may also be influenced via organ crosstalk mechanisms: inflammatory mediators, accumulation of metabolic products/uremic toxins, neuroendocrine disorders, and endothelial dysfunction can all lead to the impairment of the heart, lungs, liver, intestines, and brain, ultimately resulting in multiple organ dysfunction syndrome (Li et al., 2022; Matsuura et al., 2023). Consequently, intervening renal inflammatory responses and curtailing their dissemination has emerged as a critical concern in the prevention of urosepsis.

Presently, global protocols for the standard management of urosepsis predominantly depend on prompt surgical alleviation of blockage and the empirical intravenous delivery of broad-spectrum antibiotics. Nonetheless, antibiotic therapy encounters significant constraints: firstly, the worldwide emergence of extended-spectrum β-lactamase (ESBL)-producing and carbapenem-resistant *Escherichia coli* has resulted in a marked rise in the clinical failure rate of empirical antibiotic treatment; secondly, certain broad-spectrum antibiotics, owing to their nephrotoxicity, may elevate the risk of acute kidney injury (AKI) or exacerbate renal burden (Aslan and Akova, 2022). The disruption of gut microbiota equilibrium by broad-spectrum antibiotics may diminish the availability of beneficial metabolites, such as short-chain fatty acids, so compromising the intestinal barrier and immunological homeostasis, potentially creating a detrimental loop (Piccioni et al., 2024). Thus, urosepsis urgently needs an antibiotic-complementary approach that minimizes the negative effects of antibiotic exposure without compromising infection management, protects metabolic-immune balance, and reduces inflammatory-oxidative stress amplification. Short-chain fatty acids (SCFAs), which are mostly core metabolites made by certain anaerobic symbiotic bacteria in the gut (like *Lachnospiraceae*) through fermentation of resistant starch and dietary fiber, are important for immune system regulation and intestinal barrier integrity (Mann et al., 2024). Short-chain fatty acids have been proven to have favorable anti-inflammatory, antioxidant, anti-apoptotic, and immunomodulatory properties in a variety of disease conditions (Du et al., 2024). Butyrate acid, unlike many other short-chain fatty acids such as acetic acid and propionic acid, has substantial anti-inflammatory properties due to its position as a strong inhibitor of histone deacetylases (HDACs) and increased affinity for GPR41/43. More importantly, butyrate acid is a primary energy source for intestinal mucosal epithelial cells, directly upregulating the expression of tight junction proteins, preserving the integrity of the intestinal physical barrier, and maintaining a hypoxic intestinal environment by altering intestinal epithelial metabolism, inhibiting intestinal flora imbalance, and ensuring the stability of anaerobic beneficial bacteria (Byndloss et al., 2017). This feature, which can interfere with inflammatory signals, maintain the intestinal barrier, and regulate the gut microbiota, provides a theoretical basis for exogenous butyrate supplementation to complement antibiotic treatment in the clinical setting of urosepsis induced by antibiotic misuse.

Oxidative stress is regarded as a primary exacerbating element in inflammation-associated renal damage. Nicotinamide adenine dinucleotide phosphate (NADPH) oxidase 4 (NOX4), an enzyme present in the kidney, is essential for the regulation of oxidative stress and subsequent signaling pathways (Park et al., 2021; Liao et al., 2022). In the kidney, NOX4 overexpression is associated with increased ROS production, mitochondrial dysfunction, renal tubular epithelial cell injury, and activation of pro-inflammatory signaling pathways. Numerous investigations have found that NOX4 is involved in the pathophysiology of a variety of renal disorders (Cowley et al., 2016; Jung et al., 2016; Wang et al., 2020). Furthermore, some studies have suggested that, in the context of sepsis-associated acute kidney injury (S-AKI), inhibiting NOX4 can reduce ROS production and inhibit NF-κB activation, thereby alleviating mitochondrial damage, inflammation, and cell death (Li et al., 2023). Previous research has demonstrated that exogenous sodium butyrate supplementation can reduce renal NOX4 protein expression in a mouse model of diabetic nephropathy, indicating that it has the capacity to directly interfere in the renal NOX4-ROS axis (Ye et al., 2024). Based on this, our study posits that in urosepsis, the kidney, as the originating organ, may initiate or enhance the activation of the NF-κB signaling pathway due to elevated NOX4-ROS levels, consequently promoting inflammatory cytokine expression and immune cell infiltration, which results in the systemic dissemination of localized damage. Exogenous butyrate supplementation may downregulate NOX4 expression or diminish ROS load, potentially attenuating NF-κB activation upstream, inhibiting the propagation of inflammation, and ultimately mitigating multi-organ damage resulting from urosepsis.

Cecal ligation and puncture (CLP) is presently regarded as the gold standard in sepsis research (Buras et al., 2005; Deitch, 2005). With the initial inflammatory outbreak starting in the intestines and abdominal cavity, the CLP model basically mimics mixed multiflora overflow peritonitis brought on by lower gastrointestinal perforation. The traditional pathophysiology of clinical urosepsis is very different from this. There aren’t many standardized animal models of urosepsis, though. To mimic urosepsis brought on by urinary tract obstruction, some researchers have suggested first ligating the ureter and then injecting *E. coli* into the dilated proximal ureter 24 hours later (Cao et al., 2022). Nonetheless, this model possesses specific limitations: firstly, the two surgical procedures were conducted merely 24 hours apart, resulting in considerable harm to the experimental animals; secondly, the kidneys had already experienced 24 hours of obstructive stress prior to the bacterial injection, rendering the model akin to a secondary infectious assault superimposed on obstructive kidney injury. Consequently, our study adjusted the model to more precisely replicate the clinical scenario of ureteral obstruction resulting in urosepsis.

Accordingly, this study aimed to determine whether sodium butyrate, alone or combined with antibiotics, attenuates obstructive urosepsis in rats and whether its protective effects are associated with renal NOX4/ROS/NF-κB signaling and intestinal homeostasis. We hypothesized that butyrate would reduce urosepsis-related injury, with greater benefit when combined with antibiotics.

## 2 Materials and methods

### 2.1 Experimental model

Male Sprague-Dawley rats (250–300 g) used in the experiments were purchased from the Experimental Animal Center of Wuhan University. The animal experimental protocol was approved by the Animal Ethics Committee of Wuhan University (WP20250146). All animals were housed in a specific pathogen-free (SPF) laminar flow environment. Animal experiments were conducted in accordance with the guidelines of the National Institutes of Health (NIH) and were fully authorized by the Experimental Animal Center of Wuhan University. All animals were housed at a temperature of 27 ± 2 °C with a 12-hour light/dark cycle.

**Figure 1:**
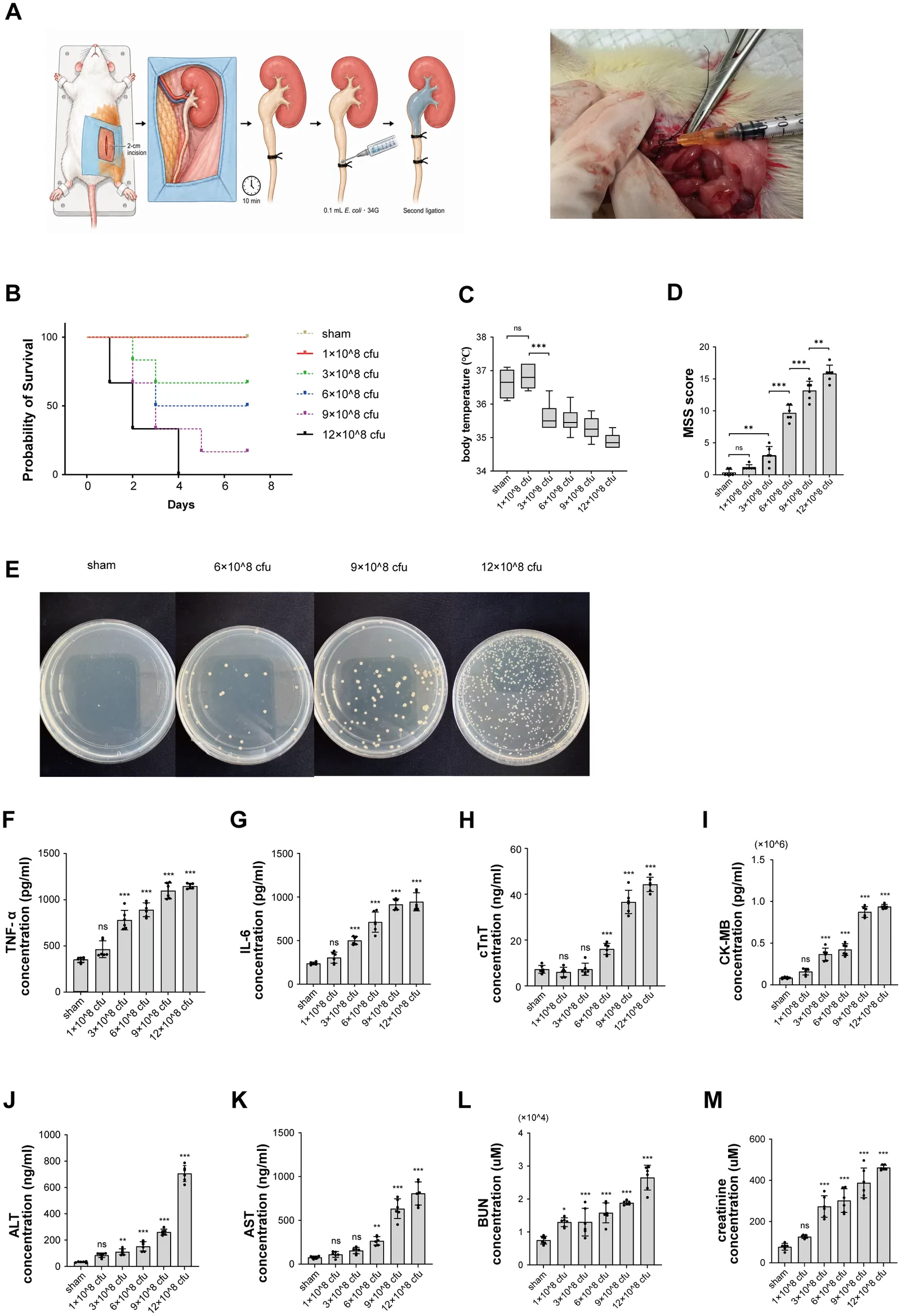
Establishment and validation of a rat model of urosepsis induced by ureteral obstruction and intrapelvic injection of *Escherichia coli*. **(A)** After ligation of the left ureter, *Escherichia coli* was injected into the dilated upper ureter. **(B)** Kaplan–Meier survival curves of rats after intrapelvic injection of different concentrations of *E. coli* (1×10^8^CFU, 3×10^8^CFU, 6×10^8^CFU, 9×10^8^CFU, 12×10^8^CFU). **(C)** Body temperature measured 24 h after modeling. **(D)** Murine Sepsis Score (MSS) at 24 h after surgery. **(E)** Representative blood culture plates from the sham, 6×10^8^CFU, 9×10^8^CFU, and 12×10^8^CFU groups. **(F, G)** Serum levels of the inflammatory cytokines TNF-α **(F)** and IL-6 **(G)**. **(H–M)** Serum biomarkers of multi-organ injury, including cardiac injury markers cTnT **(H)** and CK-MB **(I)**, liver injury markers ALT **(J)** and AST **(K)**, and renal function indices BUN **(L)** and creatinine **(M)**. Data are presented as mean ± SD; n = 6 per group. The *p*-values above the data denote statistical comparisons of the groups. ns, not significant; *p < 0.05, **p < 0.01, ***p < 0.001.

Based on earlier research, we designed one sham surgery group (laparotomy only) and five bacterial concentration gradient groups (1×10⁸CFU, 3×10⁸CFU, 6×10⁸CFU, 9×10⁸CFU, 12×10⁸CFU, respectively) (Cao et al., 2022; Hu et al., 2024), the utilized bacteria are uropathogenic *Escherichia coli* strain (BIO SCI, CFT073), 6 rats per group. After a 12-h fast, rats were anesthetized by intraperitoneal injection of freshly prepared 2.5% 2,2,2-tribromoethanol solution (Avertin; Sigma-Aldrich, St. Louis, MO, USA) at a dose of 250 mg/kg. After anesthesia was induced, the rat was placed and secured in the supine position. The skin of the left abdomen and left groin was sterilized with povidone-iodine and covered with drapes. A longitudinal incision of 2 cm in length was performed in the left abdomen, and the left kidney along with the upper ureter was mobilized using hemostats. The left ureter was occluded with silk sutures and allowed to remain in that state for 10 minutes until the renal pelvis and upper ureter became distended. Subsequently, 0.1 ml of various doses of *Escherichia coli* solution was administered into the renal pelvis above the ligation site utilizing a 34G sterile injection needle (**Figure 1A**). The ureter was re-ligated superior to the injection site, and the needle was retracted. The left kidney was reinserted into the abdominal cavity, and the layers were sutured and sterilized. Physiological saline was supplied to the animals through a heating pad to ensure proper hydration and maintain a consistent temperature of roughly 37 °C during this process. The animals were housed in separate cages and warmed with indirect light until they had recovered from anesthesia. At 24 h after surgery, blood samples were collected from the lateral tail vein under aseptic conditions. A portion of the fresh blood was used for blood culture, while the remaining sample was centrifuged at 3000 rpm for 20 minutes in a refrigerated centrifuge. The supernatant was then collected and stored at −80 °C until further analysis. Survival was evaluated four times daily for a minimum of seven days following surgery, and cumulative survival curves were constructed utilizing the Kaplan-Meier method.

Upon verifying the effective development of the model and identifying the ideal concentration of the injected bacterial solution, we employed the same methodology to develop a urosepsis model. Thirty SDRs were randomly allocated into five groups: (1) sham group, laparotomy only; (2) urosepsis group; (3) sodium butyrate treatment group, where butyrate was administered as per prior studies (Fu et al., 2019): sodium butyrate (NaB) 200 mg/kg was intravenously injected at 1 hour and 8 hours post-surgery; (4) antibiotic treatment group, with antibiotic administration following previous studies (Zhang et al., 2007): antibiotics were administered intramuscularly at 0.5 and 12 hours post-surgery (gentamicin 8 mg/kg; metronidazole 25 mg/kg; aminobenzylpenicillin 150 mg/kg); (5) combined treatment group, where NaB was intravenously injected at 1 hour and 8 hours post-surgery, and antibiotics were intramuscularly injected at 0.5 and 12 hours post-surgery. Animals were fed on an adjustable schedule until sacrifice. At 24 h after surgery, rats were anesthetized, after which blood samples were collected via inferior vena cava puncture. Serum was separated by centrifugation and stored at −80 °C until further analysis. Fresh kidney, terminal ileum, heart, and liver tissues were collected, as well as fresh rat excrement. The rats were euthanized by cervical dislocation. An independent cohort of 30 rats was assigned to the same five groups (n = 6 per group), received the same interventions, and was monitored four times daily for 7-day survival. Cumulative survival curves were generated using the Kaplan–Meier method.

### 2.2 The murine sepsis score (MSS) system

Sepsis severity was evaluated using the Murine Sepsis Score (MSS) according to published criteria (Shrum et al., 2014). The score comprised seven observational domains: appearance, level of consciousness, activity, response to stimulus, eye condition, respiratory rate, and respiratory quality. Each domain was graded from 0 to 4, with higher scores indicating more severe clinical deterioration. The total MSS for each animal was calculated by summing the seven individual domain scores, yielding a possible range of 0–28. Scoring was performed in a blinded manner at the indicated time points to provide a semiquantitative assessment of sepsis severity.

### 2.3 Microbiome analysis

Fresh fecal samples were immediately stored at −80 °C until DNA extraction. Genomic DNA was extracted using the DNeasy PowerLyzer PowerSoil Kit and was assessed by 1% agarose gel electrophoresis. The V3–V4 hypervariable region of the bacterial 16S rRNA gene was amplified using primers 341F and 806R. Based on preliminary electrophoretic quantification, PCR products were quantified using the QuantiFluor™-ST blue fluorescence quantitative system (Promega, Madison, WI, USA) and pooled according to the required sequencing amount for each sample. Libraries of purified PCR products were constructed using the NEXTFLEX Rapid DNA-Seq Kit, and sequencing was performed on the Illumina NextSeq 2000 platform (Shanghai Meiji Bio-pharmaceutical Technology Co., Shanghai, China). After demultiplexing, raw reads were quality-filtered using fastp (v0.19.6) and merged using FLASH (v1.2.11). Amplicon sequence variants (ASVs) were inferred using the DADA2 plugin in the QIIME2 workflow (version 2020.2) with recommended parameters. To reduce the influence of sequencing depth on α-diversity and β-diversity analyses, all samples were rarefied to 20,000 sequences per sample, which yielded an average Good’s coverage of 97.90%. Taxonomic assignment of ASVs was performed using the naive Bayes ++ consensus taxonomyclassifier implemented in QIIME2 against the SILVA 16S rRNA database (v138). Alpha diversity was evaluated using the Chao1 richness and Shannon diversity indices. Overall differences among the five experimental groups were assessed using the Kruskal–Wallis test, followed by Dunn’s *post hoc* test for pairwise comparisons. P values from the *post hoc* comparisons were adjusted using the Benjamini–Hochberg procedure. Beta diversity was assessed by principal coordinates analysis (PCoA) based on Bray–Curtis dissimilarities, and differences in microbial community composition among groups were tested using permutational multivariate analysis of variance (PERMANOVA). Linear discriminant analysis effect size (LEfSe) was used to identify differentially abundant taxa in two targeted comparisons: the urosepsis versus butyrate groups and the urosepsis versus combined treatment groups.

### 2.4 SCFA measurements

Fecal SCFA profiles were analyzed by ¹H-NMR spectroscopy. Fecal sediment was bead-milled in ice-cold D_2_O (5 m/s, 40 s) and then centrifuged at 16,000 × g for 5 minutes at 4 °C. The resulting fecal supernatant was filtered through an Amicon Ultra ultrafiltration membrane with a 3 kDa molecular weight cutoff and buffered with 200 mM Na_3_PO_4_/D_2_O and 5.0 mM DSS/D_2_O (pH 7.0) before transfer to 3-mm NMR tubes. Spectra were acquired at 298 K on a 600 MHz Bruker AVANCE III HD NMR spectrometer equipped with a cryogenic probe. Spectral processing and metabolite quantification were performed using Chenomx NMR Suite v8.4, with DSS used as the internal standard.

### 2.5 Histological evaluation

Kidney, liver, and ileum tissues were harvested, fixed in 4% buffered formaldehyde, dehydrated, embedded in paraffin, and sectioned at 4 μm. Sections were then subjected to hematoxylin and eosin staining. Histological changes were examined under a light microscope (DM2500; Leica, Germany) at ×200 or ×400 magnification. Tissue injury was evaluated in a blinded manner by two independent observers who were unaware of group allocation. Renal tubular injury was scored according to the percentage of tubular damage, including brush border loss, tubular dilatation/flattening, tubular degeneration, tubular cast formation, and vacuolization. Injury scores of 0, 1, 2, 3, and 4 corresponded to 0%, <25%, 26%–50%, 51%–75%, and >76% renal tubular damage, respectively (Havasi and Borkan, 2011). Intestinal injury was assessed using the Chiu scoring system (Fu et al., 2019). Liver histopathological changes, including cytoplasmic damage, vacuolization, nuclear condensation, nuclear pyknosis, nuclear depigmentation, and cell arrest, were evaluated as previously described (Yang et al., 2025).

### 2.6 Immunohistochemical assay

Paraffin-embedded kidney tissues were sectioned at 4 µm. After deparaffinization in xylene and rehydration through graded alcohols, antigen retrieval was performed by microwave heating in 0.01 M trisodium citrate. Sections were incubated overnight at 4 °C with primary antibodies against NOX4 (Proteintech, 1:100) and CD68 (Santa Cruz, 1:50). After washing with PBS, sections were incubated with a biotin-labeled secondary antibody at room temperature for 30 minutes. Random fields of the renal cortex were imaged under an Olympus BX51 microscope (×200).

### 2.7 Measurement of ROS

DHE staining was used to assess ROS generation in kidney tissue. DHE was dissolved in dimethyl sulfoxide (DMSO) at a stock concentration of 10 mM and diluted with PBS to a final concentration of 20 μM before use. Kidney tissue specimens embedded in optimal cutting temperature compound (OCT) were washed three times with PBS. After incubation with DHE (Sigma-Aldrich, St. Louis, MO, USA) solution in a light-protected humidified chamber at room temperature for 30 minutes, slides were counterstained with DAPI (4′,6-diamidino-2-phenylindole) and immediately imaged using a fluorescence microscope (DMi8, Leica).

### 2.8 Transmission electron microscopy

Cardiac tissue samples were fixed in 2.5% glutaraldehyde (G1102, Servicebio, Wuhan, China) at 4 °C for 2–4 hours. Post-fixation was then carried out in 1% osmium tetroxide dissolved in 0.1 M phosphate buffer (pH 7.4) at room temperature for 2 hours. Samples were dehydrated through a graded ethanol series (50%, 70%, 80%, 90%, 95%, and 100%) and washed twice with 100% acetone (Sinopharm Chemical Reagent Co., Ltd., Shanghai, China) for 15 minutes each. The tissues were subsequently infiltrated with acetone and EMbed-812 resin (SPI-Chem, West Chester, PA, USA) at ratios of 1:1 for 2–4 hours, 1:2 overnight, and finally pure resin for 5–8 hours. Samples were embedded in pure EMbed-812 resin and polymerized at 60 °C for 48 hours. Ultrathin sections (60–80 nm) were cut using an ultramicrotome (Leica EM UC7, Leica Microsystems, Wetzlar, Germany), stained with 2% uranium acetate and lead citrate, and examined under a 120 kV transmission electron microscope (HT7700, Hitachi, Tokyo, Japan). Images were captured using the digital camera system connected to the microscope.

### 2.9 ELISA

Serum concentrations of BUN, creatinine, IL-6, TNF-α, ALT, AST, troponin T, CK-MB, and MCP-1 were measured using ELISA kits according to the manufacturers’ instructions. All ELISA kits were purchased from MyBioSource, Inc.

### 2.10 Western blot analysis

Kidney tissue lysates were prepared in RIPA buffer supplemented with protease and phosphatase inhibitors, and protein concentrations were determined using the BCA method (Thermo Scientific, Waltham, MA, USA). For Western blot analysis, equal amounts of total protein (50 μg) from each sample were separated by SDS-PAGE, transferred to membranes, and incubated with the following primary antibodies: IκBα (CST, 1:1000), p-IκBα (CST, 1:1000), TNF-α (Proteintech, 1:1000), IL-6 (Proteintech, 1:1000), NGAL (Proteintech, 1:10000), KIM-1 (Proteintech, 1:1000), p65 (Proteintech, 1:2000), p-p65 (Proteintech, 1:10000), NOX4 (Proteintech, 1:1000), ZO-1 (Proteintech, 1:10000), and occludin (CST, 1:1000). β-actin (GeneTex, 1:1000) was used as the loading control. Protein bands were visualized using the Bio-Rad electrophoresis system and quantified with Image Lab 3.0 software. Band intensities were normalized to β-actin. Full, uncropped images of the Western blots presented in **Figures 3C, 4E**, and **5A** are provided in **Supplementary Material Figures S1–S3**, respectively.

### 2.11 Quantitative RT-PCR (RT-qPCR)

Total RNA was extracted from kidney tissue using Trizol reagent (Invitrogen, Cat. no. 15596018). cDNA (total RNA 2 μg) was synthesized using Moloney mouse leukemia virus reverse transcriptase (Promega, Madison, WI, USA). The levels of IL-6, TNF-α, MCP-1, KIM-1, NGAL, and NOX4 in kidney tissue were quantitatively detected using TaqMan real-time PCR. Primer sequences are as follows:IL-6, forward 5’-GCCCACCAGGAACGAAAGTC-3’, reverse 5’- ACTGGCTGGAAGTCTCTTGCG-3’; TNF-α, forward 5’- GCCTCTTCTCATTCCTGCTT-3’, reverse 5’- TGGGAACTTCTCATCCCTTTG-3’; MCP-1, forward 5’- TAGCATCCACGTGCTGTCTC-3’, reverse 5’- TGCTGCTGGTGATTCTCTTG-3’; KIM-1, forward 5’- CTGGAATGGCACTGTTGACATCC-3’, reverse 5’- GCAGATGCCAACATAGAAGCCC-3’; NGAL, forward 5’- GGGCTGTCCGATGAACTGAA-3’, reverse 5’- AATGCATTGGTCGGTGGGAA-3’; NOX4, forward 5’- TGGCCAACGAAGGGGTTAAA-3’, reverse 5’- TCCTAGGCCCAACATCTGGT-3’.

### 2.12 Statistical analysis

All quantitative data are presented as the mean ± SD. Statistical analyses were performed using GraphPad Prism version 11 (GraphPad Software, San Diego, CA, USA). Comparisons among multiple groups were performed using one-way ANOVA followed by Tukey’s multiple-comparisons test, and Tukey-adjusted P values were used for *post hoc* pairwise comparisons. Survival curves were estimated using the Kaplan–Meier method and compared using the log-rank (Mantel–Cox) test. All tests were two-sided, and P < 0.05 was considered statistically significant. Statistical procedures specific to the microbiome analyses are described in Section 2.3.

## 3 Results

### 3.1 Successful establishment of the urosepsis model and determination of the optimal bacterial inoculum concentration

In order to ascertain the ideal concentration of the injected bacterial solution and to confirm the effectiveness of the modified urosepticemia model developed in this work, we designed one sham surgery group (laparotomy only) and five bacterial concentration gradient groups (1×10^8^CFU, 3×10^8^CFU, 6×10^8^CFU, 9×10^8^CFU, 12×10^8^CFU, respectively). Our findings indicate that an increased concentration of *E. coli* correlates with a heightened mortality rate in rats after 7 days. Moreover, when the bacterial concentration attained 12×10^8^CFU, rats succumbed as early as 24 hours post-surgery (**Figure 1B**). Twenty-four hours post-injection of *E. coli* or saline, we assessed the body temperature of the rats. The findings indicated that when the bacterial concentration attained 1×10^8^CFU, the rats’ body temperature remained largely unchanged. When the bacterial concentration attained 3×10^8^CFU, the rats’ body temperature commenced a decline, ultimately falling below the normal range. The temperature continued to decline as bacterial concentration increased. The hypothermia observed in the rats indicates a severe sepsis phenotype, potentially associated with circulatory anomalies (**Figure 1C**). We employed the MSS scoring system to evaluate the severity of urosepsis in rats. The MSS score markedly elevated when the bacterial concentration attained 6×10^8^CFU (**Figure 1D**). Concurrently, we conducted blood cultures on sham-operated rats and rats with bacterial concentrations of 6×10^8^CFU, 9×10^8^CFU, and 12×10^8^CFU. The findings indicated that blood cultures were positive in all three urosepsis groups, whereas the sham-operated group had negative blood cultures (**Figure 1E**). Furthermore, we employed ELISA to quantitatively assess the concentrations of blood inflammatory mediators and other biochemical markers in rats. The findings indicated that following the injection of *Escherichia coli*, TNF-α and IL-6 levels were significantly elevated in comparison to the sham-operated group (**Figures 1F, G**), suggesting a more pronounced systemic inflammatory response in the urosepsis group. Additionally, there was observable impairment in heart, liver, and kidney functions (**Figures 1H–M**), aligning with the occurrence of systemic multiple organ damage (MODS) associated with urosepsis.

The experimental results demonstrated that the injection of *E. coli* into the renal pelvis via the obstructed ureter precipitated urosepsis and resulted in multi-organ damage in rats, confirming the successful establishment of the model. To confirm that the rats displayed urosepsis symptoms following modeling and to assure their survival within 24 hours post-surgery, we established the appropriate injection concentration of *E. coli* at 9×10^8^CFU.

### 3.2 Butyrate attenuated sepsis severity and systemic inflammation and was associated with improved survival in rats with urosepsis

**Figure 2:**
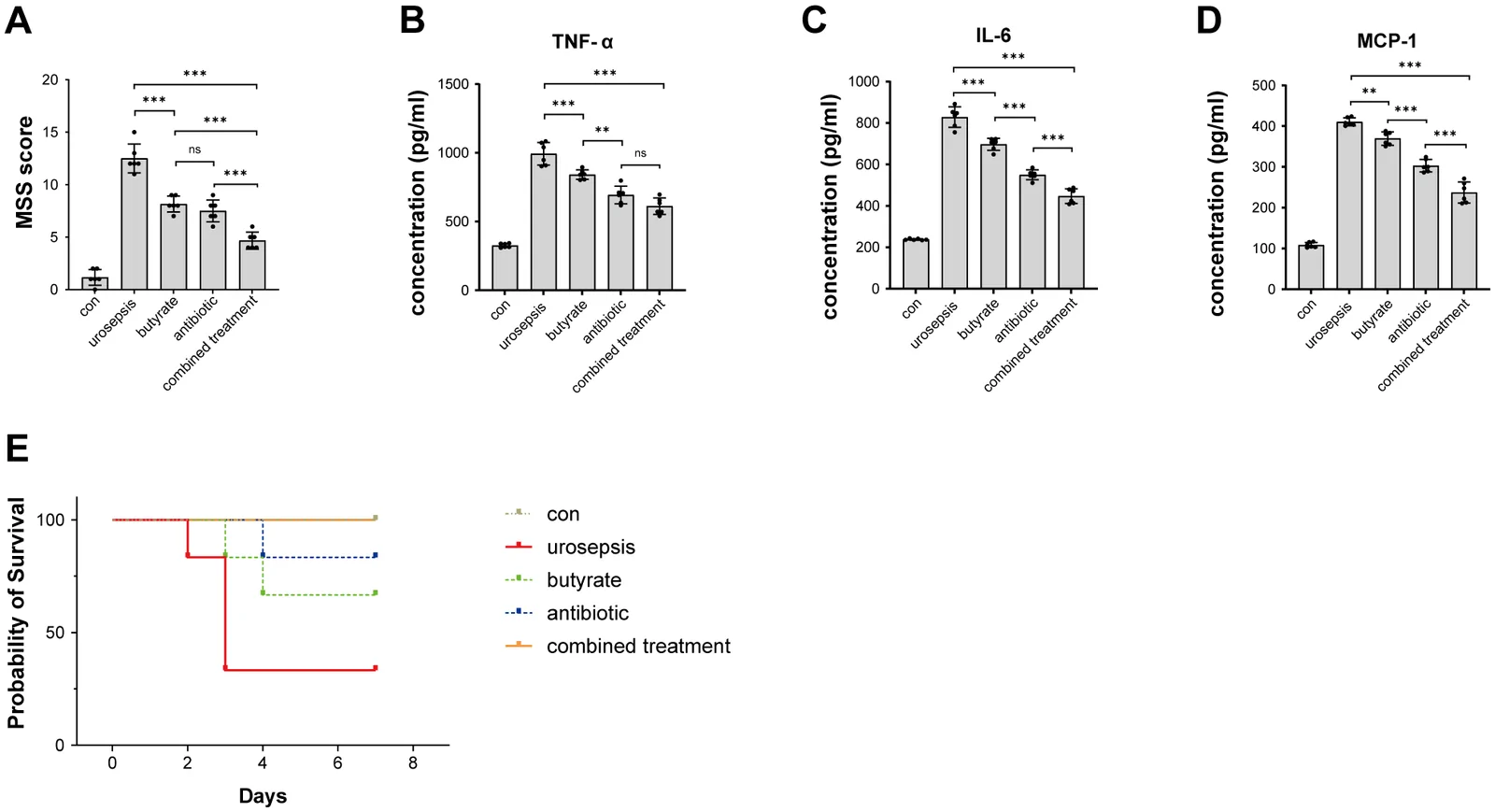
Butyrate attenuated sepsis severity and systemic inflammation and was associated with improved 7-day survival in rats with urosepsis. **(A)** Murine Sepsis Score (MSS) in rats 24 h after surgery. **(B-D)** Serum levels of TNF-α **(B)**, IL-6 **(C)**, and MCP-1 **(D)** in rats. **(E)** Kaplan–Meier survival curves for an independent cohort monitored for 7 days after surgery; n = 6 animals per group. Data are presented as mean ± SD; n = 6 per group. The *p*-values above the data denote statistical comparisons of the groups. ns, not significant; *p < 0.05, **p < 0.01, ***p < 0.001.

We established a model in rats using the ideal injection concentration of *E. coli*. The rats in the urosepsis group had typical symptoms of generalized deterioration 24 hours after modeling, including decreased activity, lethargy, piloerection/coiling, and sluggishness, in line with earlier experimental results when compared to the sham-operated group, their MSS scores rose noticeably. On the other hand, in terms of urosepsis phenotype, the butyrate treatment group displayed a declining trend in scores, which is consistent with the antibiotic treatment group. More improvement was seen in the combination treatment group (**Figure 2A**). The findings indicate that butyrate can significantly ameliorate the symptoms of urosepsis in rats, with a more pronounced impact when administered in conjunction with antibiotics compared to its use alone or with antibiotics alone. To investigate the efficacy of butyrate in mitigating the systemic inflammatory response induced by urosepsis in rats, we quantitatively measured cytokine levels in rat serum via ELISA 24 hours post *E. coli* injection. The findings indicated that, in contrast to the urosepsis group, the serum levels of pro-inflammatory cytokines (TNF-α and IL-6) and the chemokine (MCP-1) in the butyrate group were markedly diminished. No statistically significant difference was observed between the butyrate group and the antibiotic group, while the reduction of inflammatory factors was more pronounced in the combination therapy group, with values approaching those of the sham-operated group (**Figures 2B–D**). The general trend aligned with the MSS score. Finally, to determine whether these acute improvements were accompanied by differences in survival, an independent cohort was monitored for 7 days. Survival was 33.3% in the urosepsis group, compared with 66.7% and 83.3% in the butyrate and antibiotic groups, while all animals survived in the combined treatment groups (**Figure 2E**).

Taken together, these findings indicate that butyrate treatment attenuated systemic inflammation and reduced disease severity in rats with urosepsis, while combined butyrate and antibiotic treatment produced the greatest overall improvement and was associated with the highest observed survival among the treatment groups.

### 3.3 Butyrate can mitigate renal injury and localized renal inflammation resulting from urosepsis

**Figure 3:**
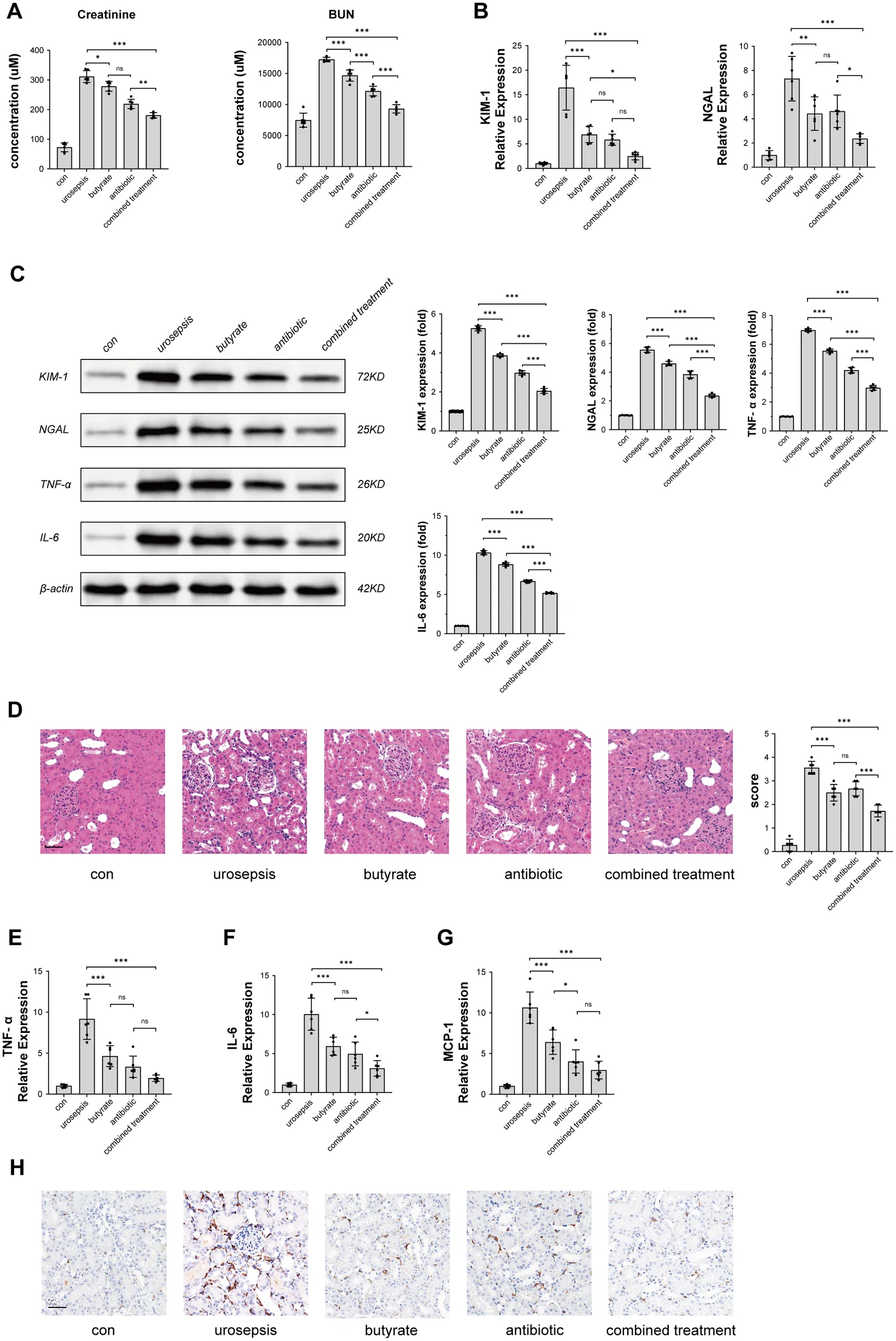
Butyrate attenuated renal injury and localized renal inflammation in rats with urosepsis. **(A)** Serum creatinine and blood urea nitrogen (BUN) levels in the sham, urosepsis, butyrate, antibiotic, and combined treatment groups. **(B)** Relative mRNA expression levels of the acute kidney injury markers KIM-1 and NGAL in kidney tissue. **(C)** Western blot analysis of KIM-1, NGAL, TNF-α, and IL-6 in kidney tissue, Protein band intensities quantified using optical densitometry and normalized to β-actin levels within the respective experimental groups. **(D)** Representative hematoxylin - eosin (H&E)-stained kidney sections and pathological injury scores. For histopathological scoring in **(D)**, three sections were randomly selected from each rat, and their scores were averaged to obtain one value per animal. Data are presented as the mean ± SD (n = 6 rats per group). Scale bars are 50 μm. **(E-G)** Relative mRNA expression levels of the inflammatory mediators TNF-α **(E)**, IL-6 **(F)**, and MCP-1 **(G)** in kidney tissue. **(H)** Representative immunohistochemical staining of CD68 in kidney tissue. Scale bars are 50 μm. Data are presented as mean ± SD; n = 6 per group. Representative images are shown in **(C, D, H)**. The *p*-values above the data denote statistical comparisons of the groups. ns, not significant; *p < 0.05, **p < 0.01, ***p < 0.001.

To ascertain if butyrate exerts a protective influence on renal function *in vivo*, we assessed serum creatinine and blood urea nitrogen levels in rats 24 hours post-modeling. The findings indicated that, in contrast to the uroseptic group, the butyrate treatment group had reduced serum creatinine and blood urea nitrogen levels, together with a notable enhancement in renal function, while the combined treatment demonstrated a more pronounced protective effect on renal function (**Figure 3A**). Real-time quantitative PCR was employed to identify the established high-sensitivity indicators of acute kidney injury, KIM-1 and NGAL, in renal tissue. The findings indicated that the expression levels of KIM-1 and NGAL were markedly reduced in the butyrate group relative to the uroseptic group, with a more pronounced decrease observed in the combination therapy group (**Figure 3B**). Western blot examination of KIM-1 and NGAL concentrations in rat kidney lysates exhibited a same trend (**Figure 3C**). To further validate that butyrate can mitigate kidney damage resulting from ureteral obstruction and infection *in vivo*, we investigated the role of butyrate via histological investigation. HE staining revealed pronounced tubular epithelial vacuolation, interstitial edema, and increased interstitial spaces in the uroseptic group, whereas these pathological alterations were markedly diminished in the butyrate group, antibiotic group, and combined therapy group (**Figure 3D**).

Inflammation at the locus of renal tissue damage is a contributor to renal function deterioration. We conducted Western blot research on pro-inflammatory cytokines (TNF-α and IL-6) and chemokines (MCP-1) in renal tissue. The findings indicated that butyrate significantly diminished local kidney inflammation, exhibiting efficacy akin to antibiotic treatment, whereas the combination therapy yielded the most favorable outcomes (**Figure 3C**). RT-qPCR further revealed decreased mRNA levels of TNF-α, IL-6, and MCP-1, with the combined treatment producing the greatest overall reductions in these inflammatory markers (**Figures 3E–G**). In alignment with the increase in inflammatory mediator production, CD68 immunohistochemistry labeling indicated that butyrate therapy markedly diminished macrophage infiltration in the kidney (**Figure 3H**).

The outcomes of the aforementioned tests indicated that in ureteral obstruction worsened by infection, butyrate might mitigate renal tissue structural damage, suppress inflammatory transcription and macrophage infiltration, and enhance renal function. Significantly, the combination treatment demonstrated a more pronounced recovery trajectory across various indices.

### 3.4 Butyrate can mitigate other organ damage resulting from urosepsis

**Figure 4:**
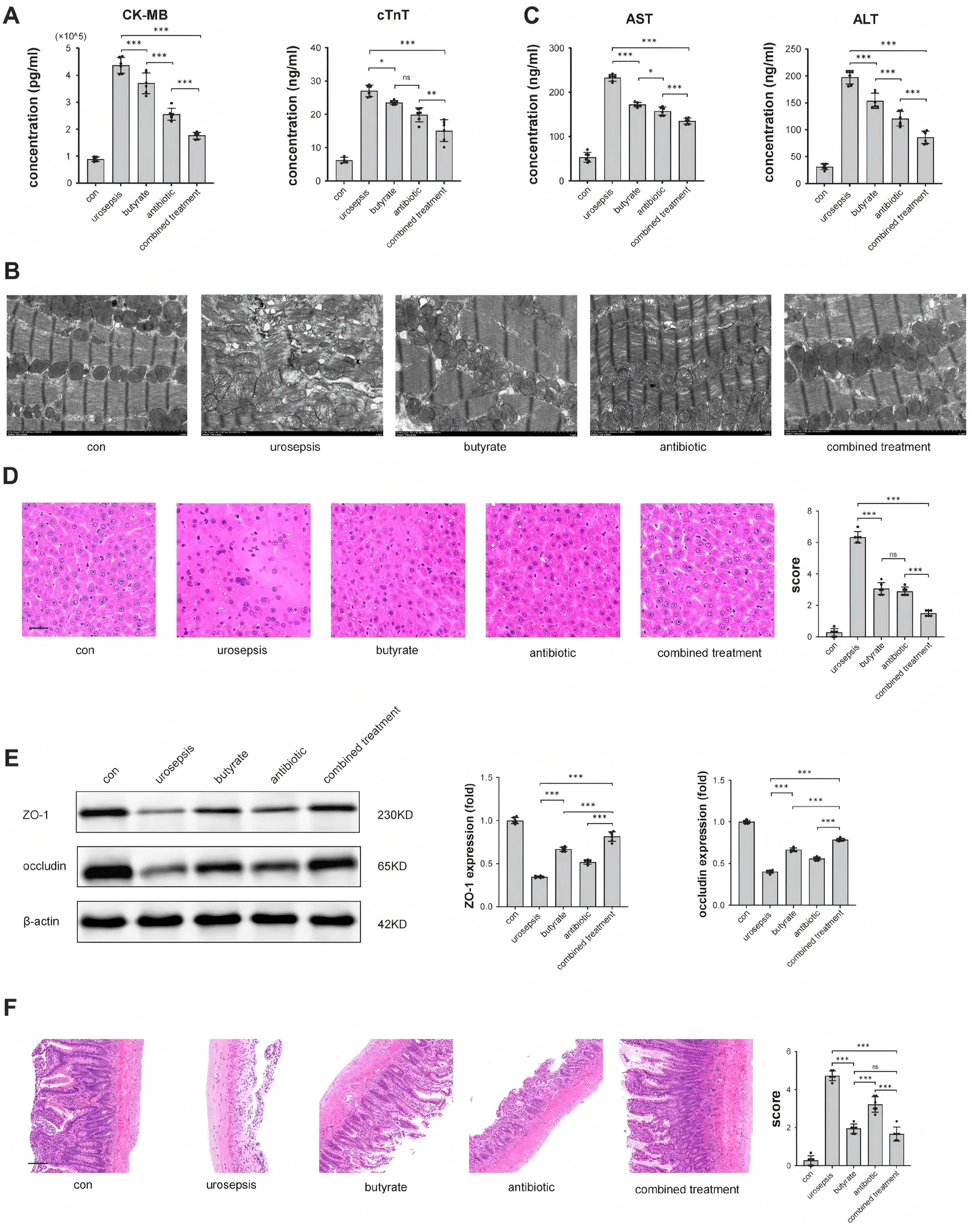
Butyrate attenuated distant organ injury and preserved intestinal barrier integrity in rats with urosepsis. **(A)** Serum levels of the cardiac injury markers CK-MB and cTnT in the sham, urosepsis, butyrate, antibiotic, and combined treatment groups. **(B)** Representative transmission electron microscopy images of myocardial ultrastructure. **(C)** Serum levels of the liver injury markers AST and ALT. **(D)** Representative hematoxylin - eosin (H&E)-stained liver sections and liver injury scores. Scale bars are 25 μm. **(E)** Western blot analysis of the tight junction proteins ZO-1 and occludin in intestinal tissue. **(F)** Representative H&E-stained terminal ileum sections and intestinal injury scores. Scale bars are 50 μm. three sections were randomly selected from each rat for each assessed tissue, and their scores were averaged to obtain one value per animal. Data are presented as the mean ± SD (n = 6 rats per group). Representative images are shown in **(B, D, F)**. The *p*-values above the data denote statistical comparisons of the groups. ns, not significant; *p < 0.05, **p < 0.01, ***p < 0.001.

Urosepsis is fundamentally a dysregulation of the host response induced by infection, resulting in potentially fatal organ failure. To examine if butyrate could mitigate multi-organ damage (except the kidneys) induced by urosepsis, we evaluated cardiac, hepatic, and intestinal function 24 hours post-model installation. Initially, we assessed the concentrations of cardiac damage indicators, specifically CK-MB and troponin T, in rat serum. The findings indicated that, in comparison to the urosepsis group, butyrate-treated rats exhibited considerably reduced serum cardiac damage biomarkers, however the reduction was less marked than that observed in the antibiotic group. The combined treatment group had the most significant reduction in heart damage biomarkers (**Figure 4A**). Electron microscopy demonstrated the ultrastructure of rat myocardium: in contrast to the control group, rats with urosepticemia displayed myocardial fiber rupture, enlarged and disorganized mitochondria, diminished mitochondrial structure, vacuolation, and the fragmentation and loss of cristae. The pathological alterations were markedly diminished in the butyrate group, although no substantial myocardial injury was seen in the combined treatment group (**Figure 4B**). The liver, serving as the body’s detoxifying center, is the primary organ impacted by urosepticemia. ELISA was employed to quantify serum ALT and AST levels, with their overall patterns paralleling those of myocardial damage markers (**Figure 4C**). HE staining of liver tissue corroborated this trend: The urosepsis group exhibited cytoplasmic vacuolization, hepatocellular necrosis, sinusoidal congestion, and inflammatory cell infiltration, whereas the butyrate group demonstrated diminished pathological damage, and the combined treatment group’s tissue architecture resembled that of the sham-operated group (**Figure 4D**). The functionality of the intestinal barrier directly indicates the presence of intestinal injury. Western blot examination of tight junction proteins ZO-1 and occludin in intestinal tissue revealed that the butyrate group had significantly elevated levels of these proteins compared to the uroseptic group. Intestinal ZO-1 and occludin levels were significantly lower in the antibiotic group than in the butyrate group, whereas the combined treatment group showed significantly higher levels of both proteins than the antibiotic group (**Figure 4E**). These findings were further confirmed by HE staining. The urosepsis group exhibited marked destruction of villous architecture, focal exposure of the lamina propria, and prominent inflammatory cell infiltration within the lamina propria and mucosa, whereas no evident histopathological damage was observed in either the butyrate-treated group or the combined treatment group (**Figure 4F**).

The present results demonstrate that both butyrate and antibiotics can mitigate distant organ damage resulting from urosepsis, while the combination treatment has a more pronounced therapeutic effect. Moreover, compared with antibiotic treatment alone, the addition of butyrate was associated with greater preservation of intestinal barrier integrity.

### 3.5 Butyrate can suppress NOX4 expression, thereby diminishing ROS generation and decreasing the activation of the NF-κB pathway

**Figure 5:**
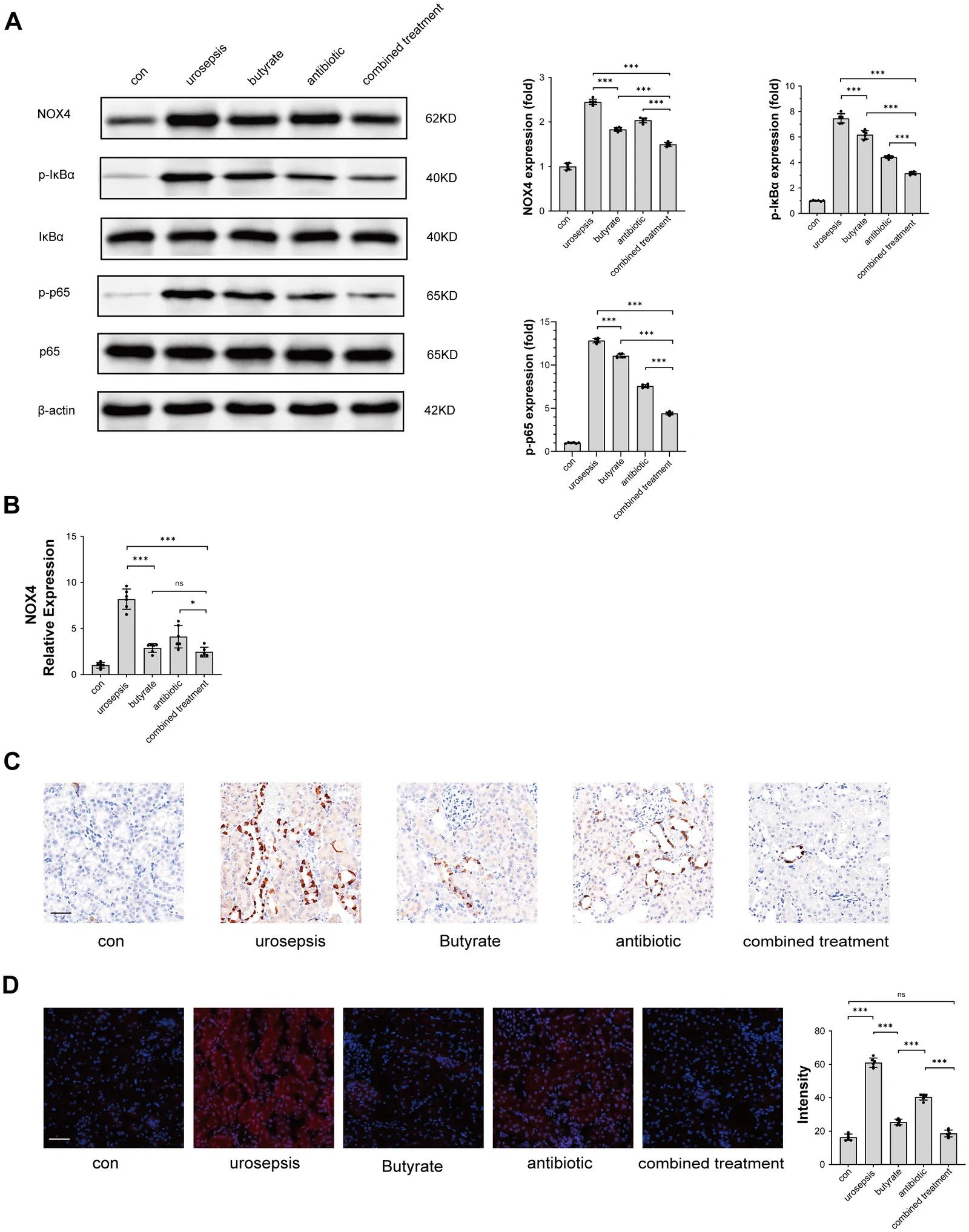
Butyrate suppressed NOX4 expression, reduced ROS production, and inhibited activation of the NF-κB pathway in the kidneys of rats with urosepsis. **(A)** Western blot analysis of NOX4 and NF-κB pathway-related proteins, including p-IκBα, IκBα, p-p65, and p65, in kidney tissue from the sham, urosepsis, butyrate, antibiotic, and combined treatment groups. **(B)** Relative NOX4 mRNA expression in kidney tissue, as determined by real-time quantitative PCR. **(C)** Representative immunohistochemical staining of NOX4 in kidney tissue. Scale bars are 50 μm. **(D)** Representative dihydroethidium (DHE) staining showing reactive oxygen species (ROS) levels in kidney tissue. Scale bars are 50 μm. Data are presented as mean ± SD; n = 6 per group. Representative images are shown in **(A, C, D)**. The *p*-values above the data denote statistical comparisons of the groups. ns, not significant; *p < 0.05, **p < 0.01, ***p < 0.001.

Based on our previous experience demonstrating that butyrate can reduce systemic inflammatory responses and multi-organ damage caused by urosepsis, and that butyrate combined with antibiotics was more effective than antibiotics alone, we were eager to learn more about butyrate’s mechanism of action in urosepsis. We first employed Western blotting to determine NOX4 expression levels in rat kidney tissue. The study found that NOX4 levels in the kidneys of rats with urosepsis were considerably higher than in the Sham group, and butyrate successfully suppressed this increase (**Figure 5A**). NOX4 mRNA levels in kidney tissue were measured using real-time quantitative PCR, and the findings revealed a similar pattern (**Figure 5B**). Additionally, NOX4 immunohistochemical staining examination produced same outcomes (**Figure 5C**). We assessed the amount of ROS in kidney tissue in order to better understand how butyrate influences urosepsis through NOX4. The butyrate group’s red fluorescence intensity, which indicates ROS levels in the kidneys, was considerably lower than that of urosepsis rats (**Figure 5D**). NOX4 functions as an upstream regulatory protein within the NF-κB signaling pathway (Park et al., 2021), the reduction in NOX4 expression will unequivocally impede the activation of the NF-κB signaling pathway. We corroborated this conclusion using Western blot analysis: relative to urosepsis, the butyrate group exhibited suppression of IkBa phosphorylation, resulting in a reduction of phosphorylated p65 levels (**Figure 5A**).

In conclusion, these findings demonstrate that in rats with ureteral blockage resulting in urosepsis, the kidney, as the source organ, undergoes markedly increased oxidative stress levels, triggering an inflammatory cascade. Butyrate treatment was associated with reduced renal NOX4 expression and oxidative stress, together with attenuated systemic inflammation and multi-organ injury.

### 3.6 Butyrate reshapes the gut microbiota

**Figure 6:**
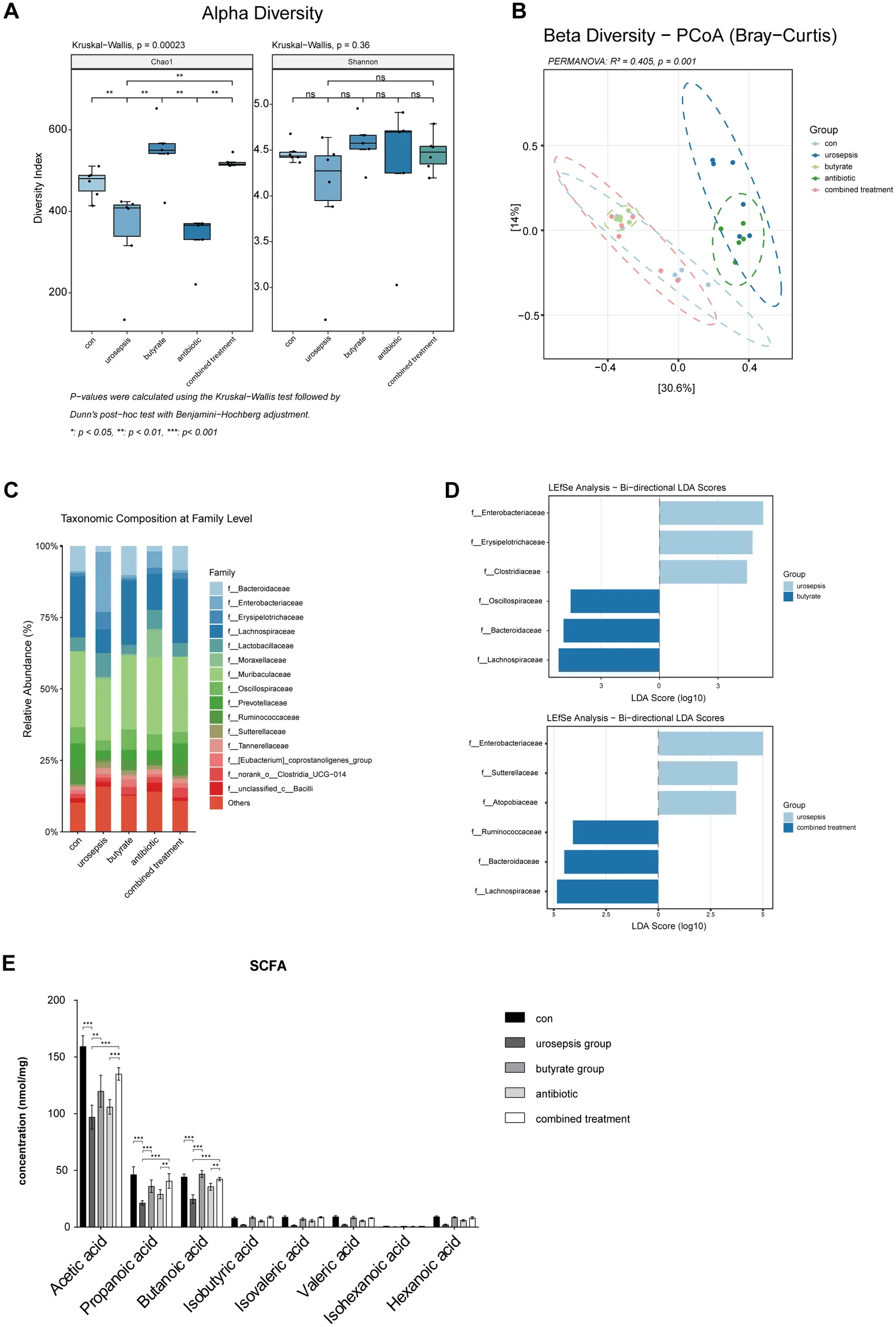
Butyrate reshaped the gut microbiota and increased short-chain fatty acid production in rats with urosepsis. **(A)** Alpha diversity assessed using the Chao1 richness and Shannon diversity indices in the sham, urosepsis, butyrate, antibiotic, and combined treatment groups. Overall differences were evaluated using the Kruskal–Wallis test, followed by Dunn’s *post hoc* test with Benjamini–Hochberg adjustment for pairwise comparisons. The overall Kruskal–Wallis P values were 0.00023 for the Chao1 index and 0.36 for the Shannon index. **(B)** Principal coordinates analysis (PCoA) based on Bray–Curtis dissimilarities. Differences in microbial community composition among the five groups were assessed using PERMANOVA (R² = 0.405, P = 0.001). **(C)** Taxonomic composition of the gut microbiota at the family level. **(D)** Bidirectional linear discriminant analysis effect size (LEfSe) scores identifying differentially abundant taxa between the urosepsis and butyrate groups (upper panel) and between the urosepsis and combined treatment groups (lower panel). **(E)** Fecal concentrations of short-chain fatty acids (SCFAs) in the five experimental groups. Each SCFA was analyzed separately using one-way ANOVA followed by Tukey’s multiple-comparisons test. SCFA data are presented as mean ± SD; n = 6 biologically independent animals per group. Adjusted pairwise comparisons are indicated as follows: ns, not significant; *P < 0.05, **P < 0.01, and ***P < 0.001.

Butyrate serves as the primary energy substrate for intestinal epithelial cells and is crucial for sustaining intestinal homeostasis and modulating the composition of beneficial microbiota (Snodgrass and Velayudhan, 2026). We conducted 16S rRNA sequencing to examine the impact of butyrate on the gut flora. The Chao1 indices indicated a considerable enhancement in the richness of the gut microbiota in the butyrate group compared to both the urosepsis group and the antibiotic group (**Figure 6A**). Moreover, Principal coordinates analysis (PCoA) based on the Bray–Curtis distance revealed that a considerable separation of the urosepsis group from both the butyrate group and the antibiotic group on PCoA, with the samples of the butyrate group exhibiting greater clustering (**Figure 6B**). This suggests that butyrate can reinstate the reduced gut microbiota diversity resulting from urosepsis, reform the gut microbiota, and mitigate gut microbiota dysbiosis. Our subsequent investigation revealed that at the family level, *Enterobacteriaceae* were markedly enriched in the urosepsis group, but the relative abundance of *Bacteroidaceae* and *Lachnospiraceae* was significantly elevated in both the butyrate and combination treatment groups (**Figure 6C**). Consistent with the family-level compositional patterns, LEfSe analysis showed that *Lachnospiraceae* and *Bacteroidaceae* were enriched in both the butyrate and combined treatment groups relative to the urosepsis group, whereas *Enterobacteriaceae* was enriched in the urosepsis group in both comparisons (**Figure 6D**). Through intestinal fermentation, the gut microbiota can create short-chain fatty acids, which are crucial for immunological modulation. Compared with the urosepsis group, the butyrate and combined treatment groups showed an overall trend toward higher fecal SCFA concentrations, with fecal butyrate showing the most pronounced increase (**Figure 6E**).

The appeal results demonstrate that urosepsis causes considerable dysbiosis and alterations in community structure, whereas butyrate enhances diversity and community structure, facilitates the restoration of short-chain fatty acid-producing gut probiotics, and subsequently elevates the levels of short-chain fatty acids in rats.

## 4 Discussion

This study evaluated critical endpoints 24 hours post-modeling in rats with urinary tract blockage. The model group demonstrated strong systemic inflammatory responses, characterized by increased serum levels of TNF-α, IL-6, and MCP-1, alongside deteriorating sepsis clinical scores. The butyrate intervention diminished these inflammatory markers and enhanced illness scores. The kidney, as the origin of infection and inflammation, exhibited substantial structural damage within 24 hours, including increased levels of inflammatory transcription and overexpression of kidney injury markers KIM-1 and NGAL. Butyrate markedly decreased renal inflammation and structural impairment while enhancing indications of renal function. The model group exhibited cardiac ultrastructural damage, hepatocyte vacuolar degeneration, and mucosal injury in the terminal ileum at the level of distant organs. Exogenous butyrate supplementation demonstrated a continuous tendency in mitigating multi-organ damage induced by urosepsis, while concurrently safeguarding intestinal barrier integrity and gut microbiota. These acute protective effects were accompanied by higher observed 7-day survival in the treatment groups than in the untreated urosepsis group, with all animals in the combined treatment group surviving.

The accuracy of animal models directly influences the viability of converting preclinical research results into human applications. The CLP model has long been regarded as the benchmark for fundamental sepsis research. The CLP model, however, results in significant peritonitis due to the translocation of various bacteria from the lower gastrointestinal tract, rather as the primary causative factor of urosepsis, which is upper urinary tract obstruction and the dissemination and exacerbation of intrarenal pelvic infection. Thus far, Hu and Cao et al. have undertaken initial investigations on urosepsis models (Cao et al., 2022; Hu et al., 2024). Nonetheless, these investigations possess specific limitations. Our research enhanced these investigations by mitigating the harm inflicted on animals throughout experimental procedures. Simultaneously, by creating various bacterial concentration gradients and monitoring postoperative survival rates and biochemical indices, we identified the optimal bacterial concentration for modeling, thereby ensuring the successful establishment of the urosepsis model and the survival of rats during the post-modeling observation period.

Current international critical care guidelines and the European Association of Urology clinical guidelines emphasize the need for prompt surgical intervention together with the early empirical intravenous administration of broad-spectrum antibiotics in the treatment of urosepsis (Rudd et al., 2020). It is important to note that the current definition of sepsis describes it as life-threatening organ dysfunction caused by a dysregulated host response to infection. Accordingly, the primary goal of treatment is to reduce the pathogen burden while simultaneously limiting the self-amplifying cycle of host response-mediated injury (Evans et al., 2021). Treatment protocols focused exclusively on bacterial eradication are facing growing clinical limitations and risks. The emergence of antimicrobial resistance has substantially undermined the efficacy of empirical antibiotic therapy. Recent multicenter epidemiological data have shown a marked increase in the detection of extended-spectrum β-lactamase-producing *Escherichia coli* and carbapenem-resistant *Enterobacterales* in patients with urosepsis, a condition associated with high mortality. In this context, multidrug resistance has become a major obstacle to the effective treatment of septic shock (Tandogdu et al., 2024). Secondly, broad-spectrum antibiotic administration can rapidly disrupt the composition of the gut microbiota and diminish the availability of microbiota-derived metabolites, thereby impairing immunometabolic regulatory signaling, particularly that mediated by short-chain fatty acids. Furthermore, in severe cases, such disruption may exacerbate dysregulation of the gut microbiota and immune axis, thereby contributing to the development of secondary infections (Shang et al., 2024). The Surviving Sepsis Campaign has explicitly highlighted the importance of early antimicrobial therapy and recommends its immediate administration within 1 hour in patients with a high suspicion of septic shock or a high probability of sepsis. Nevertheless, dynamic reassessment and the prompt discontinuation of unnecessary antibiotic exposure are also emphasized so that the benefits of treatment may be balanced against the associated trade-offs (Evans et al., 2021). Consequently, identifying adjuvant intervention techniques that integrate source-targeted inflammation management with ecological restoration is essential for mitigating the detrimental effects of sepsis on the body.

Short-chain fatty acids, as major metabolites derived from the gut microbiota, have also been recognized as important signaling molecules in host immune regulation. A large body of immunological evidence has shown that inflammatory responses can be modulated by SCFAs through G protein coupled receptors, including Ffar2/GPR43, while immune cell phenotypes and effector gene transcription can also be regulated through histone deacetylase inhibition. Accordingly, SCFAs have been shown to exert anti-inflammatory and pro resolution effects in multiple models of inflammation related diseases (Maslowski et al., 2009). Butyrate, in particular, is widely recognized as the most potent natural histone deacetylase inhibitor among the short-chain fatty acids (Eslick et al., 2022). Precise biochemical quantitative analyses have shown that butyrate inhibits HDAC with an IC50 of approximately 52 µM, whereas propionate exhibits a much higher IC50 of 223 µM (Waldecker et al., 2008), indicating that butyrate has markedly greater inhibitory potency against HDAC than propionate. By mediating HDAC inhibition at the epigenetic level, butyrate has been shown not only to suppress the transcription of proinflammatory factors, but also to downregulate CCL2 (MCP-1) and VCAM-1 expression, thereby attenuating macrophage infiltration (Aguilar et al., 2014). Furthermore, butyrate has been reported to modulate macrophage polarization by influencing the interaction between HDAC3 and STAT1, thereby promoting STAT1 acetylation and inhibiting STAT1 dimer phosphorylation. As a result, macrophage polarization is shifted toward the M2 phenotype, which is characterized by tissue reparative and anti-inflammatory properties (Zhao et al., 2023). In our study, butyrate supplementation in rats with urosepsis was associated with significant reductions in the serum levels of TNF-α, IL-6, and MCP-1, while the infiltrative density of CD68-positive cells was also markedly reversed and decreased. These findings reflect the potent anti-inflammatory effects of butyrate.

The persistently high clinical mortality associated with urinary sepsis is no longer attributable solely to local suppurative infection of the urinary tract or transient acute kidney injury, but is instead largely driven by uncontrolled systemic inflammatory response syndrome (SIRS) and subsequent MODS resulting from immune dysregulation (Li et al., 2022). In this process, kidney injury often acts not only as the initiating event but also as an amplifier of inflammation. Injured renal tubular epithelial cells release chemokines and damage associated molecular patterns (DAMPs), thereby promoting myeloid cell infiltration and intensifying local inflammatory responses. At the same time, impaired renal function disrupts internal homeostasis, facilitates the accumulation of toxic metabolites, and enhances oxidative stress, which together further aggravate injury in distant organs (Matsuura et al., 2023). In recent years, accumulating evidence from leading academic journals has emphasized that organ dysfunction in sepsis is not an isolated event, but rather a progressive cascade in which local immune dysregulation is amplified into systemic collapse through inter-organ crosstalk across inflammatory, metabolic, endothelial microcirculatory, and immune effector networks (Komaru et al., 2024; Lelubre and Vincent, 2018). Under the combined effects of mechanical ureteral obstruction and high concentrations of bacterial endotoxin, acute injury is induced in renal tubular epithelial cells (TECs), which normally mediate filtration and reabsorption, while large numbers of M1 proinflammatory macrophages are recruited into the renal interstitium in response to chemotactic signals. In this context, both cell populations undergo phenotypic transformation and metabolic reprogramming, thereby becoming sustained sources of proinflammatory mediators released into the circulation, including TNF-α, IL-6, and IL-1β, as well as highly toxic uremic solutes such as indoxyl sulfate and p-cresyl sulfate. Together, these processes drive the persistent systemic release of inflammatory mediators. Meanwhile, gut derived protein bound uremic toxins, including indoxyl sulfate and p-cresyl sulfate, can accumulate markedly in the circulation as a result of impaired proximal tubular secretory transport caused by downregulation of the organic anion transporter axis, thereby further exacerbating systemic inflammation and organ injury (Liu et al., 2018; Kounatidis et al., 2024; O'Neill and Pearce, 2016; Bush et al., 2020). Against this backdrop of near complete systemic collapse, butyrate was identified in the present study as a critical interventional node with a pronounced capacity to halt disease propagation. Early exogenous supplementation not only protected renal tubular epithelial cells, but also suppressed the sustained transcription of inflammatory cytokines and chemokines, in injured renal tissue, thereby limiting the spread of toxic mediators and disrupting the molecular signaling basis of systemic inter organ crosstalk. This framework also provides a mechanistic explanation for the experimental findings, in which butyrate intervention not only directly protected the primary target organ, but also promoted the parallel recovery of histological integrity and metabolic function in distant parenchymal organs.

NOX4, a member of the NADPH oxidase family, is highly expressed in the kidney and has been closely associated with reactive oxygen species generation and inflammatory activation in multiple models of kidney disease (Li et al., 2023; Meng et al., 2018). Previous studies have shown that butyrate may improve mitochondrial function and suppress NOX4 expression through activation of the AMPK/SIRT1/PGC-1α signaling pathway (MaChado et al., 2012). In the context of urosepsis, renal oxidative stress should not be regarded merely as a byproduct of inflammation, but rather as a pathogenic driver that is closely linked to inflammatory signaling pathways. On the one hand, excessive ROS can initiate chain reactions of lipid peroxidation in cellular and mitochondrial membrane phospholipids, thereby causing oxidative damage to mitochondrial DNA. This process further increases the likelihood of mitochondrial permeability transition pore opening under conditions of calcium overload and oxidative stress. Sustained opening of the mitochondrial permeability transition pore results in the collapse of the mitochondrial membrane potential and proton gradient, uncoupling of oxidative phosphorylation, and aggravation of ATP depletion, ultimately leading to decompensated energy metabolism (Su et al., 2019); On the other hand, ROS serves as a highly effective intracellular second messenger that facilitates activation of the IKK complex, which in turn rapidly phosphorylates IκBα (Herb et al., 2019), Activation of the NF-κB signaling pathway can upregulate a broad range of proinflammatory genes and cell death related programs, thereby establishing a self-amplifying positive feedback loop. The findings of the present study were consistent with involvement of this pathway. In the urosepsis group, renal NOX4 protein expression and ROS levels were increased, and phosphorylation of IκBα and p65 was elevated. Butyrate treatment was associated with reduced NOX4 expression, reduced ROS levels, and decreased phosphorylation of IκBα and p65. These coordinated molecular changes were consistent with modulation of the renal NOX4/ROS/NF-κB axis and were accompanied by attenuated renal inflammation and injury and reduced multi-organ damage. Within the framework of modern critical care medicine, the gut is widely recognized as the largest immune organ and microecological reactor in the body, as it harbors not only the most extensive mucosal immune system, namely gut associated lymphoid tissue (GALT), but also a highly metabolically active microbial community (Putignani et al., 2014). Numerous studies in critical care medicine have shown that, during critical illness, the intestinal barrier and gut microbiota are rapidly disrupted, and this imbalance is closely associated with systemic inflammation, immune dysregulation, and organ dysfunction (Otani and Coopersmith, 2019; Kain et al., 2024). In the present study, urosepsis was associated with marked intestinal mucosal injury, as evidenced by widespread villus atrophy and reduced expression of the tight junction proteins ZO-1 and occludin, indicating substantial disruption of epithelial barrier integrity. Such impairment of the intestinal barrier may facilitate the translocation of luminal pathogens and endotoxin-containing microbial products across the lamina propria into the systemic circulation via the portal venous system, thereby amplifying systemic inflammatory responses. Notably, butyrate treatment preserved the expression of ZO-1 and occludin and alleviated intestinal barrier damage, suggesting a protective effect on gut barrier function in the setting of urosepsis. This interpretation is supported by previous studies in which butyrate was shown to strengthen epithelial barrier integrity by promoting tight junction assembly and enhancing the junctional localization of ZO-1 and occludin, while increasing transepithelial electrical resistance and reducing paracellular permeability (Peng et al., 2009). In addition, MUC2 gene expression can also be induced by butyrate through AP-1, and histone acetylation at the MUC2 promoter in intestinal epithelial goblet cells can be promoted, thereby enhancing mucin secretion and protecting epithelial cells (Burger-van Paassen et al., 2009). Butyrate is not only the primary energy source for colonic epithelial cells (Roediger, 1982), but also a pleiotropic biochemical signaling molecule that functions within a highly synergistic network with the gut microbiota (Byndloss et al., 2017; Lange et al., 2023). Butyrate activates the peroxisome proliferator activated receptor γ signaling pathway in the host cell nucleus, thereby driving mitochondrial oxidative phosphorylation in intestinal epithelial cells, significantly increasing epithelial oxygen consumption, and maintaining the physiologically hypoxic luminal microenvironment (Byndloss et al., 2017). This hypoxic barrier restricts the diffusion of oxygen into the intestinal lumen, thereby preserving the anaerobic ecological niche and suppressing the abnormal expansion of facultative anaerobes. In the present study, the effects of butyrate on the gut microbiota were investigated by 16S rRNA sequencing analysis. Alpha diversity analysis and principal coordinate analysis based on Bray–Curtis distance showed that the gut microbiota was reshaped by butyrate treatment, with microbial diversity being maintained and community dispersion being reduced. These findings suggest that butyrate intervention partially reversed urosepsis associated gut dysbiosis and contributed to the maintenance of microbial community homeostasis. Notably, an increased relative abundance of *Lachnospiraceae* and *Bacteroidaceae* was observed after butyrate treatment, whereas *Enterobacteriaceae* was found to be significantly more abundant in the urosepsis group than in the treatment group. *Lachnospiraceae* is widely regarded as an important anaerobic taxon associated with the butyrate producing network (Rivière et al., 2016; Louis and Flint, 2009), the butyrate produced by these bacteria helps maintain physiological epithelial hypoxia through the PPAR-γ pathway, thereby suppressing the expansion of facultative anaerobic opportunistic bacteria and promoting microbial community homeostasis. *Bacteroides*, as an important component of the gut microbiota (Zafar and Saier, 2021), with a large and diverse repertoire of polysaccharide utilization loci (PULs) and carbohydrate active enzymes (CAZymes), *Bacteroides* is equipped to mediate the initial degradation and acquisition of complex dietary glycans and represents a major source of propionate (Shkoporov and Hill, 2025). Furthermore, previous studies have shown that *Bacteroides* can synergistically promote the proliferation of butyrate producing bacteria through cross feeding. Specifically, complex glycans are cleaved by upstream *Bacteroides* through polysaccharide utilization loci, leading to the release of shared substrates and key intermediates such as acetate. These substrates and acetate are subsequently utilized by downstream butyrate producing bacteria to support their proliferation and butyrate synthesis, thereby enhancing the overall butyrate producing capacity of the microbial community (Culp and Goodman, 2023). An increased abundance of facultative anaerobic *Enterobacteriaceae* is often regarded as an ecological signal of inflammation (Kain et al., 2024). Inflammation can alter the redox environment of the intestinal lumen and increase the availability of electron acceptors such as nitrate, thereby conferring a respiratory advantage on *Enterobacteriaceae* in the inflamed gut and promoting their expansion (Winter et al., 2013). In the setting of barrier disruption, *Enterobacteriaceae* associated pathogen associated molecular patterns are more likely to translocate into the circulation, thereby enhancing the production of proinflammatory mediators such as TNF-α, IL-6, and MCP-1 through activation of the TLR4-MyD88/TRIF-NF-κB pathway and, at least in the short term, exacerbating renal tubular stress and local inflammatory infiltration. Therefore, butyrate appears to play a critical role in preserving intestinal barrier integrity, microbial homeostasis, and immune balance during urosepsis.

Our findings underscore the translational potential of butyrate based intervention in the context of urosepsis. Although early intravenous administration of broad spectrum antibiotics remains the cornerstone of current treatment for urosepsis, the accompanying crisis of antimicrobial resistance and microbiota depletion has emerged as a major limitation of this approach. In the present study, combined treatment with butyrate and antibiotics was shown to outperform antibiotic monotherapy across multiple dimensions in rats, including serum inflammatory cytokine levels, indicators of multiorgan function, tissue architecture, and gut microbial ecological balance. Taken together, these findings suggest a potentially valuable therapeutic strategy for future urosepsis management, in which early antibiotic mediated pathogen reduction is integrated with butyrate mediated immunomodulation and restoration of gut microbial homeostasis. Antibiotics enable the rapid clearance of high pathogen burdens in the systemic circulation and infected tissues, whereas butyrate modulates the immune network, selectively suppresses the renal NOX4/ROS/NF-κB cascade, and preserves gut microbial homeostasis. This combined strategy may, in the future, reduce the dependence on high dose broad spectrum antibiotics during the early phase of empirical treatment for urosepsis and shorten the duration of antibiotic exposure. Moreover, by interrupting the crosstalk that drives multiorgan dysfunction, it may ultimately improve overall survival and promote higher quality recovery of multiorgan function in patients with urosepsis.

Although beneficial effects of butyrate intervention were observed in the context of urosepsis, several limitations of the present study should be acknowledged. First, although survival was monitored for 7 days in a separate cohort, the biochemical, histological, mechanistic, microbiome, and SCFA outcomes were assessed only at the acute 24-h time point. Therefore, the temporal evolution of organ injury and the effects of butyrate on longer-term organ repair remain to be determined. Second, although reduced NOX4 expression, decreased ROS accumulation, and suppression of NF-κB signaling were observed, the precise mechanism by which butyrate downregulates NOX4 expression remains to be elucidated. Third, current preclinical studies of the gut microbiota rely predominantly on rodent hosts, which may not fully recapitulate the complexity of the human microbiome or the specific interactions among microbial taxa. Fourth, although NaB was administered twice, at 1 and 8 h after surgery, only one dose level of 200 mg/kg per administration was evaluated. This dose was selected *a priori* based on a previously published rat study (Fu et al., 2019); however, the absence of a dose-ranging design precluded evaluation of dose-dependent effects and determination of the minimum effective or optimal dose in obstructive urosepsis.

In conclusion, based on a urosepsis model induced by ureteral obstruction combined with bacterial inoculation into the ureter, the present study demonstrated that butyrate attenuated systemic inflammation and alleviated multiorgan injury, with the kidney being the principal target organ affected. These protective effects were accompanied by reduced renal NOX4 expression, reduced ROS levels, and decreased NF-κB pathway activation, as well as preservation of intestinal barrier integrity and attenuation of gut microbiota dysbiosis. Collectively, these findings suggest that butyrate may serve as an effective adjunct to antibiotic therapy and highlight its translational potential as a therapeutic strategy for urosepsis.

## Data Availability

The datasets presented in this study can be found in online repositories. The names of the repository/repositories and accession number(s) can be found below: https://www.ncbi.nlm.nih.gov/, PRJNA1474614.
