# Supplementary material for: Butyrate mitigates multi-organ damage in urosepsis: associated changes in the renal NOX4/ROS/NF-κB axis and gut microbiota
Source: Front Cell Infect Microbiol. 2026 Sep 1;16:1912506. doi: 10.3389/fcimb.2026.1912506 (PMC13574636; doi:10.3389/fcimb.2026.1912506)
Supplement: Supplementary file 1 [file SupplementaryFile1.docx]

**Supplementary Materials**

This document contains the available full, uncropped source images for the representative Western blots presented in Figures 3C, 4E, and 5A of the main manuscript. Images are reproduced without cropping or alteration. In each image, the sample lanes are arranged from left to right as sham, urosepsis, butyrate, antibiotic, and combined treatment, followed by the molecular-weight marker.

**Contents**

Supplementary Figure S1 (corresponding to Figure 3C): KIM-1, NGAL, TNF-α, IL-6, and β-actin.

Supplementary Figure S2 (corresponding to Figure 4E): ZO-1, occludin, and β-actin.

Supplementary Figure S3 (corresponding to Figure 5A): NOX4, IκBα, p-IκBα, p65, p-p65, and β-actin.

# Supplementary Figure S1A

**Full, uncropped KIM-1 Western blot corresponding to main-text Figure 3C.**

Supplementary Figure S1A. Full, uncropped KIM-1 image corresponding to Figure 3C. The sample lanes are ordered from left to right as sham, urosepsis, butyrate, antibiotic, and combined treatment; the molecular-weight marker is shown at the right.

# Supplementary Figure S1B

**Full, uncropped NGAL Western blot corresponding to main-text Figure 3C.**

Supplementary Figure S1B. Full, uncropped NGAL image corresponding to Figure 3C. The sample lanes are ordered from left to right as sham, urosepsis, butyrate, antibiotic, and combined treatment; the molecular-weight marker is shown at the right.

# Supplementary Figure S1C

**Full, uncropped TNF-α Western blot corresponding to main-text Figure 3C.**

Supplementary Figure S1C. Full, uncropped TNF-α image corresponding to Figure 3C. The sample lanes are ordered from left to right as sham, urosepsis, butyrate, antibiotic, and combined treatment; the molecular-weight marker is shown at the right.

# Supplementary Figure S1D

**Full, uncropped IL-6 Western blot corresponding to main-text Figure 3C.**

Supplementary Figure S1D. Full, uncropped IL-6 image corresponding to Figure 3C. The sample lanes are ordered from left to right as sham, urosepsis, butyrate, antibiotic, and combined treatment; the molecular-weight marker is shown at the right.

# Supplementary Figure S1E

**Full, uncropped β-actin loading-control blot corresponding to main-text Figure 3C.**

Supplementary Figure S1E. Full, uncropped β-actin loading-control image corresponding to Figure 3C. The sample lanes are ordered from left to right as sham, urosepsis, butyrate, antibiotic, and combined treatment; the molecular-weight marker is shown at the right.

# Supplementary Figure S2A

**Full, uncropped ZO-1 Western blot corresponding to main-text Figure 4E.**

Supplementary Figure S2A. Full, uncropped ZO-1 image corresponding to Figure 4E. The sample lanes are ordered from left to right as sham, urosepsis, butyrate, antibiotic, and combined treatment; the molecular-weight marker is shown at the right.

# Supplementary Figure S2B

**Full, uncropped occludin Western blot corresponding to main-text Figure 4E.**

Supplementary Figure S2B. Full, uncropped occludin image corresponding to Figure 4E. The sample lanes are ordered from left to right as sham, urosepsis, butyrate, antibiotic, and combined treatment; the molecular-weight marker is shown at the right.

# Supplementary Figure S2C

**Full, uncropped β-actin loading-control blot corresponding to main-text Figure 4E.**

Supplementary Figure S2C. Full, uncropped β-actin loading-control image corresponding to Figure 4E. The sample lanes are ordered from left to right as sham, urosepsis, butyrate, antibiotic, and combined treatment; the molecular-weight marker is shown at the right.

# Supplementary Figure S3A

**Full, uncropped NOX4 Western blot corresponding to main-text Figure 5A.**

Supplementary Figure S3A. Full, uncropped NOX4 image corresponding to Figure 5A. The sample lanes are ordered from left to right as sham, urosepsis, butyrate, antibiotic, and combined treatment; the molecular-weight marker is shown at the right.

# Supplementary Figure S3B

**Full, uncropped IκBα Western blot corresponding to main-text Figure 5A.**

Supplementary Figure S3B. Full, uncropped IκBα image corresponding to Figure 5A. The sample lanes are ordered from left to right as sham, urosepsis, butyrate, antibiotic, and combined treatment; the molecular-weight marker is shown at the right.

# Supplementary Figure S3C

**Full, uncropped p-IκBα Western blot corresponding to main-text Figure 5A.**

Supplementary Figure S3C. Full, uncropped p-IκBα image corresponding to Figure 5A. The sample lanes are ordered from left to right as sham, urosepsis, butyrate, antibiotic, and combined treatment; the molecular-weight marker is shown at the right.

# Supplementary Figure S3D

**Full, uncropped p65 Western blot corresponding to main-text Figure 5A.**

Supplementary Figure S3D. Full, uncropped p65 image corresponding to Figure 5A. The sample lanes are ordered from left to right as sham, urosepsis, butyrate, antibiotic, and combined treatment; the molecular-weight marker is shown at the right.

# Supplementary Figure S3E

**Full, uncropped p-p65 Western blot corresponding to main-text Figure 5A.**

Supplementary Figure S3E. Full, uncropped p-p65 image corresponding to Figure 5A. The sample lanes are ordered from left to right as sham, urosepsis, butyrate, antibiotic, and combined treatment; the molecular-weight marker is shown at the right.

# Supplementary Figure S3F

**Full, uncropped β-actin loading-control blot corresponding to main-text Figure 5A.**

Supplementary Figure S3F. Full, uncropped β-actin loading-control image corresponding to Figure 5A. The sample lanes are ordered from left to right as sham, urosepsis, butyrate, antibiotic, and combined treatment; the molecular-weight marker is shown at the right.
